# Sphingolipids in Inflammation: From Bench to Bedside

**DOI:** 10.1155/2016/7602526

**Published:** 2016-02-24

**Authors:** Kazuyuki Kitatani, Kazuhisa Iwabuchi, Ashley Snider, Laura Riboni

**Affiliations:** ^1^Tohoku Medical Megabank Organization/Department of Obstetrics and Gynecology, Tohoku University, 2-1 Seiryo-machi, Aoba-ku, Sendai 980-8573, Japan; ^2^Institute for Environmental and Gender Specific Medicine, Juntendo University Graduate School of Medicine, Chiba, Japan; ^3^Stony Brook Cancer Center, Stony Brook University, Stony Brook, NY, USA; ^4^Northport Veterans Affairs Medical Center, Northport, NY, USA; ^5^Department of Medical Biotechnology and Translational Medicine, LITA-Segrate, University of Milan, Milan, Italy

Sphingolipids have been appreciated as bioactive lipids that regulate a diverse range of cellular responses [1, 2]. In recent years many efforts of researchers were made to improve our knowledge of sphingolipids in pathophysiological inflammation. Different studies demonstrated that cellular signaling in inflammatory processes is controlled by ceramide [3], sphingosine-1-phosphate (S1P) [4], ceramide-1-phosphate [5], and glycosphingolipids (such as lactosylceramide and GM3) [6]. The molecular mechanisms underlying this signaling have been extensively studied.

This special issue is composed of ten articles including three research articles and seven review articles. These contributions review important discoveries and provide novel findings that support the multifaceted role of sphingolipids in inflammation.

Dysregulated formation of several sphingolipids including S1P and ceramide has been implicated in inflammatory bowel disease (IBD). L. Abdel Hadi et al. describe sphingolipid metabolism and signaling in IBD and discuss the potential of sphingolipid-targeted molecules as therapeutic strategies for this disease.

Metabolic disease, such as obesity and type 2 diabetes, is emerging as a major health crisis in many countries. High fat diet is a primary contributing factor for obesity and its related diseases. S. Choi and A. J. Snider review the evidence for sphingolipid metabolism and pathobiology in models of high fat diet.

Glycosphingolipids cluster with sphingomyelin and cholesterol in plasma membranes, forming lipid microdomains (lipid rafts) considered as platforms for signal transduction. K. Iwabuchi et al. review the evidence for biological significance of lactosylceramide-enriched microdomains in immunological and inflammatory responses of neutrophils. They also overview the significance of ceramide species and its metabolites in biological functions. A. M. Bryan et al. discuss the findings pointing to the importance of sphingolipids in immune responses of macrophages and neutrophils to fungal infections. R. Ghidoni et al. review roles of sphingolipid in the pathobiology of lung inflammation.

Three research articles discuss novel findings for S1P and its receptors. E. Moon et al. discover an involvement of S1P in stroke damage in initial and recurrent stroke models. A. Chumanevich et al. reveal that S1P/S1P receptor 2 axis promotes mast cell angiogenic potential. C. Zhao et al. demonstrate that the sphingolipid pathway controlling S1P levels is dysregulated in rheumatoid arthritis synovial fibroblasts. In addition, M. Aoki et al. and S. Mahajan-Thakur et al. review the evidence pointing to roles of S1P and its receptors in immune system and blood coagulation system.

This special issue discusses the topics associated with sphingolipid metabolism and pathobiology in inflammation. The articles in this special issue not only provide novel findings in sphingolipid pathobiology, but also discuss the evidence collected from a large number of research articles, giving insight into drug discovery for inflammation-associated diseases.
